# Health-related quality of life in patients with progressive glioblastoma treated with combined bevacizumab and lomustine versus lomustine only: Secondary outcome of the randomized phase III EORTC 26101 study

**DOI:** 10.1093/nop/npae091

**Published:** 2024-09-25

**Authors:** Linda Dirven, Abigirl Machingura, Martin J van den Bent, Corneel Coens, Andrew Bottomley, Alba A Brandes, Julien Domont, Ahmed Idbaih, Johan A F Koekkoek, Jaap C Reijneveld, Michael Platten, Wolfgang Wick, Martin J B Taphoorn

**Affiliations:** Department of Neurology, Haaglanden Medical Center, The Hague, The Netherlands; Department of Neurology, Leiden University Medical Center, Leiden, The Netherlands; Quality of Life Department, European Organization for Research and Treatment of Cancer, Brussels, Belgium; The Brain Tumor Center, Erasmus MC Cancer Institute, Rotterdam, The Netherlands; Quality of Life Department, European Organization for Research and Treatment of Cancer, Brussels, Belgium; Quality of Life Department, European Organization for Research and Treatment of Cancer, Brussels, Belgium; Department of Medical Oncology, AUSL-IRCCS Scienze Neurologiche, Bologna, Italy; Institut Gustave Roussy, Villejuif, France; Sorbonne Université, AP-HP, Institut du Cerveau - Paris Brain Institute - ICM, Inserm, CNRS, Hôpitaux Universitaires La Pitié Salpêtrière - Charles Foix, Service de Neuro-Oncologie, F-75013, Paris, France; Department of Neurology, Haaglanden Medical Center, The Hague, The Netherlands; Department of Neurology, Leiden University Medical Center, Leiden, The Netherlands; Department of Neurology and Brain Tumour Center Amsterdam, Amsterdam University Medical Centers, Amsterdam, The Netherlands; UMM, Mannheim, Heidelberg University and CCU Neuroimmunology, Heidelberg, Germany; German Consortium of Translational Cancer Research (DKTK), Clinical Cooperation Unit Neurooncology, German Cancer Research Center, Heidelberg, Germany; Neurology Clinic and National Centre for Tumour Diseases, University Hospital Heidelberg, Heidelberg, Germany; Department of Neurology, Haaglanden Medical Center, The Hague, The Netherlands; Department of Neurology, Leiden University Medical Center, Leiden, The Netherlands

**Keywords:** bevacizumab, EORTC, glioblastoma, health-related quality of life, lomustine, patient-reported outcome, recurrent

## Abstract

**Background:**

Progression-free survival, but not overall survival, was prolonged with bevacizumab and lomustine compared to lomustine only in the randomized phase 3 European Organization for Research and Treatment of Cancer (EORTC) 26101 study.

**Objective:**

To evaluate the impact of treatment on health-related quality of life (HRQoL) in progressive glioblastoma patients participating in the EORTC 26101 study.

**Methods:**

Patients with progressive glioblastoma, after standard radio-chemotherapy, were 2:1 randomized to either BEV/LOM or LOM. HRQoL was a secondary trial outcome and assessed using the EORTC QLQ-C30 and QLQ-BN20 questionnaires at baseline, and subsequently every 12 weeks. Predefined scales for analysis were global health status (GH), physical functioning, social functioning (SF), motor dysfunction, and communication deficit. The primary endpoint was HRQoL during the last assessment up to week 36. Moreover, time to HRQoL deterioration (TTD) and HRQoL deterioration-free survival (DFS) were calculated.

**Results:**

Out of 437 patients, 402 (92%) patients had a baseline HRQoL assessment, which dropped to 66% at week 36. During the last assessment up to week 36, no differences were observed for predefined scales, apart from SF being clinically relevant lower in the combination arm (mean 66.0 versus 81.0, *p* = .001). Of note, the baseline SF score was 66.4 for patients in the combination arm, showing stable SF. Median DFS was significantly longer in the combination arm (12.4 weeks) compared to lomustine alone (6.7 weeks), reflecting the difference in time to progression between arms. TTD, not including progression as an event, was not different between treatment arms (median 13.0 versus 12.9 weeks).

**Conclusion:**

The addition of bevacizumab to lomustine did not negatively affect HRQoL during the progression-free period.

The prognosis for patients with newly diagnosed glioblastoma, the most common and severe malignant primary brain tumor in adults,^1^ remains poor.^2,3^ The median overall survival with standard treatment with resection followed by concomitant chemoradiation and adjuvant chemotherapy with temozolomide is <15 months.^4^ Inevitably, patients will experience disease recurrence, for which current treatment options are scarce and the effectiveness poor.^5,6^

Several uncontrolled studies evaluated treatment with bevacizumab, an antiangiogenic agent, which was shown to improve progression-free survival (PFS) in patients with recurrent glioblastoma.^7–9^ The Dutch BELOB study, a randomized phase 2 trial, showed that the 9-month overall survival was 43% in the lomustine monotherapy arm, 38% in the bevacizumab monotherapy arm, and 63% in the combined bevacizumab plus lomustine arm.^10^ In addition, both median progression-free and overall survival were more favorable in the combination arm compared to the monotherapy arms, with similar toxicity^10^ and impact on health-related quality of life outcomes.^11^ The results of the BELOB trial led to the initiation of the European Organization for Research and Treatment of Cancer (EORTC) 26101 randomized phase 3 trial, in which a total of 437 patients were randomized to treatment with lomustine alone (*n* = 149) or treatment with combined bevacizumab and lomustine (*n* = 288).^12^ Despite the prolonged median PFS in patients treated with combined bevacizumab plus lomustine (4.2 months versus 1.5 months; hazard ratio [HR] of 0.49 [95% CI: 0.39-0.61, *p* < .001]), overall survival was similar between the treatment arms. Indeed, patients also treated with bevacizumab had a median overall survival of 9.1 months versus 8.6 months for patients treated with lomustine alone (HR for death was 0.95, 95% CI: 0.74-1.21; *p* = .65).^12^ However, patients in the combination arm did experience more often grade 3 to 5 toxicity compared to the lomustine monotherapy arm, 63.6% versus 38.1% respectively.^12^

To determine the net clinical benefit of a treatment strategy, not only the quantity of survival should be considered but also the impact of treatment on patient-centered outcomes such as neurocognitive functioning and HRQoL. In this study, we report on the influence of combined treatment with bevacizumab and lomustine on the HRQoL of recurrent glioblastoma patients, which was a secondary endpoint of the EORTC 26101 study.

## Methods

### Study Population

Patients eligible for participation in the EORTC 26101 trial had unequivocal signs of first progression after chemoradiation for histologically confirmed glioblastoma (at least 3 months after the end of radiation). Resection for progression was allowed if performed >2 weeks prior to randomization and full recovery was established. Patients were required to have a good performance status (World Health Organization (WHO) score ≤ 2) and adequate hematological, renal, and hepatic function. Also, patients had to be on a stable or decreasing dose of steroids for seven days prior to the baseline MRI scan, and treatment with nonenzyme-inducing antiseizure medication was allowed. Further details on the study population are available elsewhere.^12^ All patients provided written informed consent, and the study was approved by the ethical review boards of all participating centers.

### Study Design and Treatment

Patients were randomized in a 1:2 ratio to treatment with lomustine alone or combined bevacizumab with lomustine. Lomustine was administered at a dose of 110 mg/m^2^ every 6 weeks. Patients in the combination group received lomustine at a dose of 90 mg/m^2^ every 6 weeks and bevacizumab at a dose of 10 mg/kg body weight every 2 weeks. Treatment was given until the second progression, after which subsequent treatment was according to the physician’s choice.

### HRQoL Assessment

The EORTC core Quality of Questionnaire (QLQ-C30, version 3.0) was used in combination with the brain cancer module (QLQ-BN20)^13–15^ Both tools were previously used in international clinical trials that investigated bevacizumab in glioma patients,^11,16^ and have shown robust psychometric properties.^13–15^ Following the EORTC scoring manual, raw item scores were aggregated and transformed into a linear scale ranging from 0 to 100.^17^ For functioning scales, a higher score represents better functioning, while for the symptom scales, a higher score represents a higher level of symptoms. A difference of at least 10 points on any scale was deemed clinically relevant.^18^

HRQoL was assessed at baseline and subsequently every 12 weeks. Time windows for acceptable HRQoL forms were applied to minimize the number of forms lost at each follow-up assessment and were set at no earlier than 2 weeks before baseline up to the day of randomization (i.e., baseline assessment) and from 1 month before and 1 month after each scheduled follow-up assessment. Forms completed outside these time windows were considered ineligible. Based on previous studies and expert opinion, five scales were selected for primary analysis: global health status (GH), physical functioning (PF), social functioning (SF), motor dysfunction (MD), and communication deficit (CD). All other scales were analyzed on an exploratory basis.

### Statistical Analysis

#### Descriptive Statistics

Descriptive statistics were used to report the sociodemographic and clinical characteristics of the patients with at least 1 HRQoL scale. Both means with standard deviations, medians, and ranges were calculated. For nominal variables, frequencies and percentages were calculated. Differences between groups were tested with a 2-sided χ^2^-test, independent *t*-test, or Mann–Whitney *U* test.

Compliance with HRQoL assessments was determined for each follow-up moment and was calculated by dividing the number of valid HRQoL forms at a specific time point by the number of expected forms at that moment.

We anticipated a more or less similar significant amount of missing data beyond the first 12 weeks as in the BELOB study.^11^ Also, differential drop out was expected between the treatment arms, as our hypothesis was that patients treated with bevacizumab would prolong PFS as compared to patients treated with lomustine. Thus, patients treated with lomustine were expected to drop out at an earlier time point than patients treated with bevacizumab.

#### HRQoL scores over time

The primary endpoint of this study was HRQoL during the last assessment. However, due to decreasing compliance over time it was decided to limit the analysis up to week 36, after which compliance was below 60%, hampering further analysis. Questionnaires completed after the moment of progression were not included in this analysis. The scores on the predefined scales were compared between treatment groups by means of Wilcoxon rank sum tests. In addition, the mean change from baseline to weeks 12, 24, and 36 was calculated. Finally, for each scale, the area under the curve (AUC) up to week 36 was calculated as the product of the scale score over a period by the duration of the period. For patients with incomplete follow-up data, the AUC was censored at the last date of follow-up. In case a patient died before week 36, the score was considered worst (0 for functioning scales and 100 for symptom scales) at all following time points. Each resulting AUC was divided by the maximum possible AUC and multiplied by 100, allowing a standardized interpretation of the AUC score in the percentage of the maximum AUC. Differences in AUC between treatment arms were compared by means of a log-rank test, and the median AUCs were calculated using the Kaplan–Meier method.

#### HRQoL postprogression

For patients who completed an HRQoL questionnaire at progression, or thereafter, we evaluated how HRQoL changed due to progression. To do so, we looked at a change in HRQoL before progression (defined as the HRQoL score on the last date before progression) to after progression (defined as the HRQoL score recorded on the first date after progression).

#### Impact of Toxicity on HRQoL Outcomes

Since the adverse event rate was higher in the combination arm, we investigated whether more severe toxicity had an impact on the selected HRQoL scales. To so do, we compared HRQoL scores at week 12 between patients with CTCAE grade 3-5 versus those without grade 3-5 toxicity. The 12-week period was chosen as the first available time point for HRQoL data (with high compliance rates) and for expected/potential toxicity due to bevacizumab and lomustine. The CTCAE grades were reported by the clinicians. Toxicity assessed within two weeks of the HRQoL assessment was considered eligible.

#### Deterioration-free survival and time to deterioration

Similar to previous studies,^16,19^ deterioration-free survival (DFS) was defined as the time from randomization until the first time a deterioration in HRQoL score ≥ 10 points (without subsequent >10-point improvement) was observed, or progressive disease or death due to any cause. Time to HRQoL deterioration (TTQD) was defined similarly to DFS, but here progression and death were excluded as events. For both analyses, patients without an event were censored at their last known HRQoL assessment. Kaplan–Meier analyses were used to calculate median DFS and TTQD, and log-rank tests were used to assess differences between treatment arms.

Several sensitivity analyses were performed, to assess the impact of the chosen time windows and missing data patterns. All analyses were performed with SAS version 9.4 (SAS Institute), and a *p* < .05 was considered statistically significant.

## Results

### Study Population

**Figure 1.**
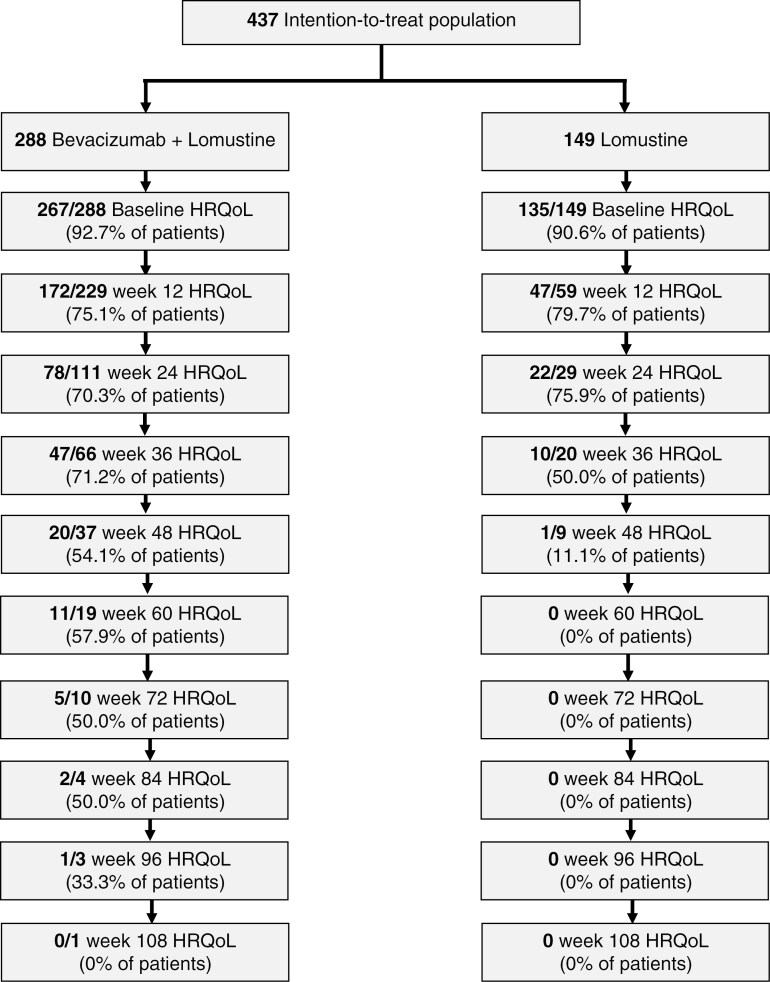
Compliance with health-related quality of life (HRQoL) assessments over time, separately for the two treatment arms. Compliance rates were calculated as the percentage of the number of HRQoL forms received divided by the expected number of forms at that time point.

Of the 437 patients randomized, 402 patients (92%) completed at least one HRQoL scale at baseline: 267/288 (92.7%) in the combination arm and 134/149 (90.6%) in the lomustine alone arm. Compliance decreased over time in both arms (Figure 1) and was below 60% after week 36. The most common reasons for noncompletion were that the patient was too ill (26.4%) and administrative failure (25.5%).

The baseline characteristics were similar between treatment arms (Table 1). Most patients were male (60%), and the median age was 57.5 years (range: 21.2-82.3). Most patients (89.9%) had WHO performance status <2, and a fair proportion used corticosteroids and antiseizure medication at baseline (50.2% and 64.9%, respectively). Only a small proportion of patients (20.1%) underwent resection for progressive disease.

**Table 1.** Baseline sociodemographic and clinical characteristics of the 402 patients in the EORTC 26101 study who had at least a valid baseline health-related quality of life form

Baseline characteristics	Lomustine (*n* = 135)	Bevacizumab + lomustine (*n* = 267)	Total (*n* = 402)
Age, years			
Median	58.5	57.2	57.5
Range	21.2-79.2	23.1-82.3	21.2-82.3
Sex, *n* (%)			
Male	83(61.5)	158 (59.2) 1	241 (60.0)
Female	52 (38.5)	09 (40.8)	161 (40.0)
WHO performance status, *n* (%)			
0	44 (32.6)	94 (35.2)	138 (34.3)
1	76 (56.3)	147 (55.1)	223 (55.5)
2	15 (11.1)	26 (9.7)	41 (10.2)
MGMT status, *n* (%)			
Methylated	35 (25.9)	59 (22.1)	94 (23.4)
Unmethylated	37 (27.4)	84 (31.5)	121 (30.1)
Undetermined/missing	63 (46.7)	124 (46.4)	187 (46.5)
Corticosteroid therapy at trial entry, *n* (%)			
No	67 (49.6)	133 (49.8)	200 (49.8)
Yes	68 (50.4)	134 (50.2)	202 (50.2)
Antiseizure medication, *n* (%)			
No	42 (31.1)	99 (37.1)	141 (35.1)
No EIAED	91 (67.4)	163 (61.0)	254 (63.2)
Switch > 2 weeks	2 (1.5)	3 (1.1)	5 (1.2)
Switch ≤ 2 weeks	0 (0.0)	1 (0.4)	1 (0.2)
EIAED	0 (0.0)	1 (0.4)	1 (0.2)
Surgery/biopsy for progression, *n* (%)			
No	111 (82.2)	210 (78.7)	321 (79.9)
Yes	24 (17.8)	57 (21.3)	81 (20.1)

Abbreviations: EIAED, enzyme-inducing antiepileptic drugs; MGMT, *O*6-methylguanine methyltransferase; WHO, World Health Organization.

### HRQoL Scores Over Time

Mean and median baseline HRQoL scores were similar between treatment arms (Table 2 for the predefined scales and Supplementary Table S1 for the exploratory scales/items). Scores for the predefined scales were worse than the scores of the general population, but also compared to the patients included in the BELOB study.^11^

**Table 2.** Mean and median baseline health-related quality of life scores for the predefined scales of the EORTC QLQ-C30 and QLQ-BN20 questionnaires

Baseline HRQoL scores	Bevacizumab + lomustine (*n* = 267)	Lomustine (*n* = 135)	All patients (*n* = 402)	General population^18^	Patient in BELOB study (*n* = 138)
EORTC QLQ-C30					
Global health status					
Median	66.7	66.7	66.7	78 (17)	75.0
Range	0-100	0-100	0-100		8.3-100
Mean (SD)	63.4 (20.6)	64.9 (21.5)	63.9 (20.9)		71.6 (19.3)
Number of patients	266	133	399		138
Physical functioning					
Median	80	86.7	86.7	90 (15)	86.7
Range	0-100	6.7-100	0-100		0-100
Mean (SD)	76.7 (23.1)	80.3 (20.6)	77.9 (22.3)		81.9 (20.3)
Number of patients	267	135	402		138
Social functioning					
Median	66.7	83.3	66.7	94 (16)	83.3
Range	0-100	0-100	0-100		0-100
Mean (SD)	66.4 (29.4)	71.2 (30.0)	68.0 (29.6)		78.1 (23.5)
Number of patients	266	133	399		137
EORTC QLQ-BN20					
Motor deficits					
Median	11.1	11.1	11.1	N/A	11.1
Range	0-100	0-77.8	0-100		0-77.8
Mean (SD)	22.2 (24.7)	14.9 (18.9)	19.8 (23.2)		18.6 (22.9)
Number of patients	267	134	401		135
Communication deficit					
Median	11.1	16.7	11.1	N/A	11.1
Range	0-100	0-100	0-100		0-100
Mean (SD)	24.4 (27.2)	25.0 (28.4)	24.6 (27.6)		21.8 (26.7)
Number of patients	266	133	399		136

**Figure 2.**
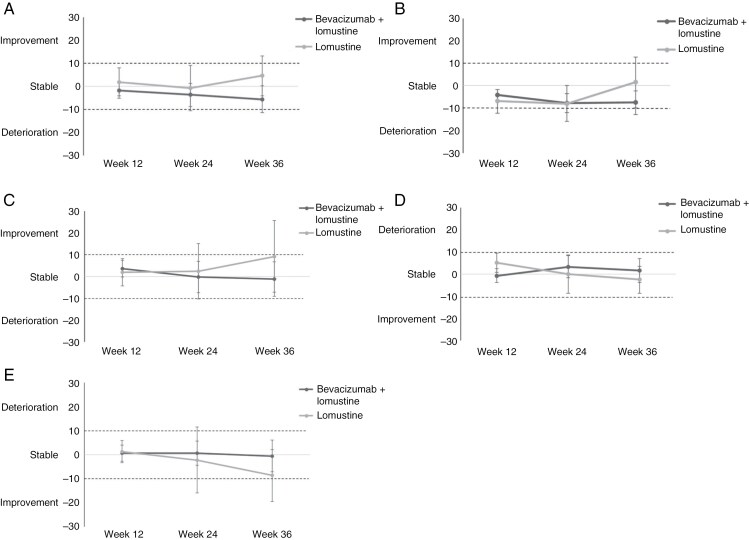
Mean changes in scores from baseline for global health status (A), physical functioning (B), social functioning (C), motor dysfunction (D), and communication deficit (E), separately for bevacizumab plus lomustine and lomustine alone. Differences in scores ≥10 points are considered clinically relevant.

The modified primary endpoint for this HRQoL analysis was the level of HRQoL during the last assessment in the follow-up period to week 36. There were no differences between treatment arms for GH status (median 66.7 versus 66.7, *p* = .198, for bevacizumab plus lomustine and lomustine alone, respectively), PF (median 75.0 versus 86.7, *p* = .210), MD (median 11.1 versus 11.1, *p* = .175) and CD (median 11.1 versus 11.1, *p* = .195). In contrast, patients treated with combined bevacizumab and lomustine had significantly and clinically worse SF compared to patients treated with lomustine alone (median 66.7 versus 100.0, *p* = .0011). It should be noted, though, that the median baseline score for SF was 66.7 in the bevacizumab + lomustine arm and 83.3 in the lomustine alone arm, showing stable SF for patients in the combination arm and improved SF for patients in the lomustine alone arm during follow-up (Figure 2C).

Mean changes in HRQoL over time for the predefined scales are presented in Figures 2A-E. The figures show that throughout the 36-week assessment period, mean changes from baseline were stable (<10-point change from baseline) for all predefined scales in both treatment arms. Results for the exploratory scales are presented in Supplementary Figure S1.

There were no statistically significant differences between treatment arms in percent AUC in any of the predefined scales. After correction for censoring, the median (95% CI) percent AUC for GH status was 34.7% (27.8-43.1%) for patients in the bevacizumab plus lomustine arm and 34.7% (25.0-70.8%) in the lomustine alone arm (*p* = .349). The median percentages for the other scales are presented in Table 3. Overall, the results indicate that patients in both treatment arms have impaired functioning and high symptom burden, as the percentages for the functioning scales range between 33.3 and 54.4% of the maximum, and between 33.3 and 42.6% for the symptom scales.

**Table 3.** Median (95% CI) percent AUC until week 36 with a correction for censoring, separately for the two treatment arms

Baseline HRQoL scores	Bevacizumab + lomustine	Lomustine	*P*
EORTC QLQ-C30			
Global health status			
Median, 95% CI	34.7 (27.8-43.1)	34.7 (25.0-70.8)	.349
Number of patients	147	31	
Physical functioning			
Median, 95% CI	37.8 (32.2-50.0)	54.4 (30.0-74.4)	.185
Number of patients	147	34	
Social functioning			
Median, 95% CI	33.3 (30.6-41.7)	36.1 (30.6-72.2)	.084
Number of patients	146	34	
EORTC QLQ-BN20			
Motor deficits			
Median, 95% CI	42.6 (33.3-55.6)	35.2 (31.5-72.2)	.083
Number of patients	145	34	
Communication deficit			
Median, 95% CI	42.6 (33.3-55.6)	33.3 (31.5-68.5)	.268
Number of patients	143	34	

Of note, the symptom scale scores were reversed in this analysis, meaning that a low score for a symptom scale represents a high level of symptomatology.

### HRQoL Postprogression

In a subset of patients, 92/288 (31.9%) in the combination arm and 31/149 (20.8%) in the lomustine arm, HRQoL data was available at the moment of progression. Compared to baseline, only PF was clinically relevant worse at progression in the combination arm (−10.2 in the combination arm versus −8.2 in the lomustine arm, Supplementary Table S2). For all other predefined scales, similarly, for both treatment arms, HRQoL deteriorated but not to a clinically relevant extent.

For 66/288 (22.9%) patients in the combination arm and 24/149 (16.1%) patients in the lomustine arm forms were available after progression, with a median of 11.4 and 10.9 weeks after progression, respectively. The change in HRQoL before and after progression was not clinically relevant for any of the predefined scales (Table 4).

**Table 4.** Mean and median changes in health-related quality of life scores for the predefined scales of the EORTC QLQ-C30 and QLQ-BN20 questionnaires before and after progression

Baseline HRQoL scores	Bevacizumab + lomustine (*n* = 66)	Lomustine (*n* = 24)	All patients (*n* = 90)
EORTC QLQ-C30			
Global health status			
Median	−8.3	−8.3	−8.3
Range	−83.3 to 41.7	−50.0 to 25.0	−83.3 to 41.7
Mean (SD)	−9.1 (24.0)	−9.8 (17.3)	−9.3 (22.3)
Number of patients	65	23	88
Physical functioning			
Median	0.0	−6.7	−6.7
Range	−73.3 to 40.0	−40 to 20	−73.3 to 40.0
Mean (SD)	−9.9 (21.8)	−5.6 (12.7)	−8.7 (19.7)
Number of patients	65	24	89
Social functioning			
Median	0.0	0.0	0.0
Range	−83.3 to 83.3	−50.0 to 50.0	−83.3 to 83.3
Mean (SD)	−5.8 (32.9)	2.9 (25.9)	−3.1 (31.2)
Number of patients	62	23	85
EORTC QLQ-BN20			
Motor deficits			
Median	0.0	0.0	0.0
Range	−88.9 to 88.9	−44.4 to 55.6	−88.9 to 88.9
Mean (SD)	4.9 (24.0)	3.3 (18.0)	4.5 (22.5)
Number of patients	64	22	86
Communication deficit			
Median	0.0	0.0	0.0
Range	−55.6 to 66.7	−22.2 to 44.4	−55.6 to 66.7
Mean (SD)	2.6 (19.3)	3.4 (17.2)	2.8 (18.7)
Number of patients	64	23	87

### Impact of Toxicity on HRQoL Outcomes

Patients with severe toxicity (CTCAE score grade 3-5) had significantly worse global health status and PF at week 12 as compared to patients without severe toxicity (Supplementary Table S3). The difference in PF was also clinically relevant (a difference of 10.1 points).

### Deterioration-Free Survival and Time to Deterioration

The addition of bevacizumab to lomustine resulted in a significant prolongation of DFS (median 12.4 versus 6.7 weeks, *p* < .001), reflecting the difference in time to progression between treatment arms. Indeed, when looking at TTQD (i.e., excluding progression as an event), there was no significant difference between treatment arms (median 13.0 versus 12.9 weeks in the bevacizumab plus lomustine and lomustine alone arm respectively, *p* = .759). This means that the treatment itself did not impact the time to HRQoL deterioration (Supplementary Figure S1).

## Discussion

The EORTC 26101 phase 3 randomized trial showed an increase in PFS in patients treated with combined bevacizumab and lomustine compared to lomustine alone, which did not translate into prolonged overall survival.^12^ This secondary analysis showed that the addition of bevacizumab to lomustine is not detrimental to HRQoL. Although we showed that SF was significantly and clinically relevant lower at the last assessment until week 36 in the combination arm compared to the lomustine alone arm, the patients in the combination arm did not deteriorate compared to baseline (mean difference in score of 1.1 points between baseline and week 36). Instead, patients in the lomustine alone arm showed an improvement in SF in week 36 compared to baseline (mean difference in score of 9.2 points), likely driven by the low number of patients available for analysis during follow-up. This result should, therefore, be interpreted with caution. Indeed, a decline in SF has been observed in previous studies in progressive glioblastoma patients. One study showed that recurrent glioblastoma patients treated with tumor treatment fields or temozolomide alone had a deterioration in SF of >5% in the first three months after randomization.^20^ In the BELOB study, only patients treated with bevacizumab alone showed a clinically relevant deterioration in SF over time, while this was not found for patients treated with lomustine alone or combined bevacizumab and lomustine.^11^ It should be noted, though, that patients in the EORTC 26101 already had worse SF at baseline compared to patients in the BELOB study (Table 2: 68.0 versus 78.1, respectively).

The addition of bevacizumab to lomustine resulted in prolonged PFS, which may be meaningful if a patient’s functioning and well-being are maintained during that period. This study showed that DFS was prolonged in the combination arm, while there was no difference in TTQD. These results, therefore, show that the addition of bevacizumab has no detrimental impact on the patient’s functioning during the progression-free period. Nevertheless, the presence of severe treatment toxicity (i.e., patients with grade 3-5 toxicity) had a negative impact on GH status and PF. Although patients in the combination arm experienced more often grade 3-5 toxicity (63.6% in the combination arm versus 38.1% in the lomustine alone arm), this difference in frequency may be explained by the longer treatment period in the combination arm.^12^

In many trials with glioma patients, information on HRQoL beyond disease progression is lacking.^16,19–26^ In this trial, we do have information on the patients’ functioning and well-being after progression in a subpopulation of patients (*n* = 123, 28.1%). These data showed that although patients had worse functioning and more symptoms after progression, these changes were not clinically relevant. Previous studies in glioma patients^27–29^ have shown that HRQoL deterioration is mainly driven by tumor growth,^30^ and not treatment. Although results are not directly comparable (i.e., data before and after progression were not compared, but only the course after progression), one other phase 3 trial collected HRQoL data after disease progression, in which patients with MGMT-unmethylated glioblastoma were randomized to treatment with bevacizumab combined with irinotecan or to treatment with temozolomide alone.^31^ This study showed that the time to postprogression deterioration was prolonged for MD and headache in the temozolomide alone arm, in which the vast majority of patients received cross-over second-line bevacizumab.^32^ The authors concluded that this trial provided indirect evidence that bevacizumab was beneficial for the maintenance of HRQoL after progression in relapsed glioblastoma. In the EORTC 26101, 48.7% of the patients received therapy after progression, of which almost half were bevacizumab. It remains unclear, however, whether this has contributed to the maintenance of HRQoL after progression in our study sample.

As in other cancer clinical trials, the major limitation of this study is the amount of missing HRQoL data, particularly during follow-up. This may have resulted in attrition bias, where patients with more favorable characteristics are overrepresented during follow-up. Although drop-out patterns did not seem to influence outcomes to a clinically relevant extent in this study (data not shown), this type of bias may explain the difference in one of our primary endpoints, i.e., SF at the last assessment up to week 36, where only one-third of the patients in the lomustine arm were included in the analysis. Moreover, as this study represents a trial population, generalization of the results to the entire recurrent glioblastoma patient population is hampered. Another issue is that many factors may impact HRQoL, such as epilepsy^33^ and comorbidity, the use of supportive treatment such as antiseizure medication^34,35^ and corticosteroids,^27^ and feelings of anxiety or depression.^36,37^ However, since this study was an RCT, the impact of these factors in HRQoL should be nondifferential (i.e., the impact is the same in both arms). Finally, other endpoints, such as neurocognition, are also important in determining the net clinical benefit of a treatment strategy. In the EORTC 26101 study, neurocognition was also measured, and it was found that the combination arm did not have poorer neurocognitive functioning over time.^12^ Nevertheless, it may be that differences in neurocognition will become apparent at longer follow-up only.

In conclusion, the EORTC 26101 study showed that the addition of bevacizumab to lomustine did result in prolonged PFS but not overall survival and that HRQoL was not negatively impacted by this treatment during the progression-free period. It is, therefore, still unclear what the standard of care for patients with progressive glioblastoma should be.^38^ Where the BRAIN trial^7^ showed that the use of bevacizumab resulted in lesser use of dexamethasone, this was not confirmed in the EORTC 26101.^12^ Bevacizumab is used in clinical practice in patients with progressive glioblastoma in order to at least maintain HRQoL during the disease course. This is supported by the findings from our study, where bevacizumab was added to lomustine and postponed further progression compared to lomustine only while maintaining patients’ functioning and well-being. One might argue that bevacizumab only could have a similar meaningful impact on HRQoL, thereby also temporarily obscuring progression. Further research in new treatment strategies continues to be warranted in this patient population, and it has been recommended that recruitment into appropriate trials should be considered for all progressive glioblastoma patients in order to improve the care of these patients.^38^

## Supplementary material

Supplementary material is available online at *Neuro-Oncology Practice* (https://academic.oup.com/nop/).

npae091_suppl_Supplementary_Material
