# Supplementary material for: Health-related quality of life in patients with progressive glioblastoma treated with combined bevacizumab and lomustine versus lomustine only: Secondary outcome of the randomized phase III EORTC 26101 study
Source: Neurooncol Pract. 2024 Sep 25;12(2):209–18. doi: 10.1093/nop/npae091 (PMC11913650; doi:10.1093/nop/npae091)
Supplement: npae091_suppl_Supplementary_Material [file npae091_suppl_supplementary_material.docx]

**Supplementary Table 1.** **Mean and median baseline health-related quality of life scores for exploratory scales of the EORTC QLQ-C30 and QLQ-BN20 questionnaires.**

| **Baseline HRQoL scores** | **Bevacizumab + Lomustine**  **(n=267)** | **Lomustine**  **(n=135)** | **All patients**  **(n=402)** | **General population^17^** | **Patient in BELOB study**  **(n=138)** |
| --- | --- | --- | --- | --- | --- |
| *EORTC QLQ-C30* |  |  |  |  |  |
| Role functioning  Median  Range  Mean (SD)  No. of patients | 66.7  0-100  63.6 (31.1)  267 | 66.7  0-100  65.6 (33.3)  135 | 66.7  0-100  64.2 (31.9)  402 | 89 (21) | 66.7  0-100  66.9 (30.3)  138 |
| Emotional functioning  Median  Range  Mean (SD)  No. of patients | 66.7  0-100  66.1 (24.7)  266 | 75  0-100  70.3 (22.7)  134 | 66.7  0-100  67.5 (24.1)  400 | 89 (16) | 75  8.3-100  73.3 (18.7)  138 |
| Cognitive functioning  Median  Range  Mean (SD)  No. of patients | 66.7  0-100  66.6 (26.4)  266 | 66.7  0-100  65.4 (27.7)  135 | 66.7  0-100  66.2 (26.8)  401 | 92 (15) | 66.7  0-100  70.8 (25.4)  138 |
| Fatigue  Median  Range  Mean (SD)  No. of patients | 33.3  0-100  37.6 (25.1)  266 | 33.3  0-100  33.9 (22.4)  135 | 33.3  0-100  36.3 (24.3)  401 | 17 (20) | 22.2  0-100  28.5 (23.6)  138 |
| Nausea and vomiting  Median  Range  Mean (SD)  No. of patients | 0  0-66.7  4.62 (10.5)  267 | 0  0-66.7  4.7 (11.6)  135 | 0  0-66.7  4.6 (10.9)  402 | 2.7 (10) | 0  0-66.7  4.1 (12.1)  138 |
| Pain  Median  Range  Mean (SD)  No. of patients | 0  0-100  16.1 (24.2)  267 | 0  0-100  12.6 (21.0)  135 | 0  0-100  14.9 (23.2)  402 | 15 (22) | 0  0-66.7  6.8 (16.2)  137 |
| Dyspnoea  Median  Range  Mean (SD)  No. of patients | 0  0-100  12.2 (22.0)  266 | 0  0-100  12.4 (23.3)  135 | 0  0-100  12.2 (22.4)  401 | 7.1 (17) | 0  0-100  6.0 (16.2)  138 |
| Insomnia  Median  Range  Mean (SD)  No. of patients | 0  0-100  26.2 (31.8)  266 | 0  0-100  26.1 (32.3)  134 | 0  0-100  26.2 (31.9)  400 | 14 (23) | 0  0-100  18.0 (27.7)  137 |
| Appetite loss  Median  Range  Mean (SD)  No. of patients | 0  0-100  10.6 (20.9)  265 | 0  0-100  8.6 (19.9)  135 | 0  0-100  9.9 (20.6)  400 | 3.3 (12) | 0  0-100  4.6 (15.7)  138 |
| Constipation  Median  Range  Mean (SD)  No. of patients | 0  0-100  15.2 (26.1)  265 | 0  0-100  8.6 (17.7)  135 | 0  0-100  13.0 (23.8)  400 | 4.8 (14) | 0  0-100  7.3 (18.8)  137 |
| Diarrhoea  Median  Range  Mean (SD)  No. of patients | 0  0-100  5.1 (14.2)  264 | 0  0-66.7  4.44 (12.8)  135 | 0  0-100  4.9 (13.7)  399 | 3.9 (14) | 0  0-66.7  6.0 (14.7)  138 |
| Financial difficulties  Median  Range  Mean (SD)  No. of patients | 0  0-100  17.2 (27.0)  263 | 0  0-100  17.9 (27.6)  134 | 0  0-100  17.5 (27.2)  397 | 3.1 (13) | 0  0-100  11.1 (22.9)  138 |
| *EORTC QLQ-BN20* |  |  |  |  |  |
| Future uncertainty  Median  Range  Mean (SD)  No. of patients | 41.7  0-100  43.1 (26.6)  267 | 33.3  0-100  40.6 (25.8)  134 | 41.7  0-100  42.2 (26.3)  401 | N/A | 33.3  0-100  36.5 (20.7)  134 |
| Visual disorders  Median  Range  Mean (SD)  No. of patients | 11.1  0-77.8  16.5 (19.9)  265 | 11.1  0-100  16.4 (20.4)  134 | 11.1  0-100  16.4 (20.1)  399 | N/A | 11.1  0-100  14.8 (19.9)  136 |
| Headache  Median  Range  Mean (SD)  No. of patients | 0  0-100  20.7 (29.7)  263 | 0  0-100  18.2 (24.8)  132 | 0  0-100  19.8 (26.9)  395 | N/A | 0  0-100  15.9 (22.9)  136 |
| Seizures  Median  Range  Mean (SD)  No. of patients | 0  0-100  3.9 (12.6)  262 | 0  0-100  5.1 (18.2)  132 | 0  0-100  4.3 (14.7)  394 | N/A | 0  0-66.7  3.0 (11.2)  134 |
| Drowsiness  Median  Range  Mean (SD)  No. of patients | 33.3  0-100  26.5 (28.7)  264 | 33.3  0-100  25.6 (24.2)  134 | 33.3  0-100  26.2 (27.2)  398 | N/A | 0  0-100  14.1 (21.3)  135 |
| Hair loss  Median  Range  Mean (SD)  No. of patients | 0  0-100  9.5 (21.4)  261 | 0  0-100  10.2 (25.1)  131 | 0  0-100  9.7 (22.7)  392 | N/A | 0  0-33.3  2.2 (8.4)  135 |
| Itchy skin  Median  Range  Mean (SD)  No. of patients | 0  0-100  11.0 (22.0)  266 | 0  0-66.7  8.5 (18.2)  133 | 0  0-100  10.2 (20.8)  399 | N/A | 0  0-66.7  8.3 (17.6)  133 |
| Weakness of legs  Median  Range  Mean (SD)  No. of patients | 0  0-100  15.6 (25.0)  263 | 0  0-100  12.2 (21.1)  134 | 0  0-100  14.4 (23.8)  397 | N/A | 0  0-100  7.9 (19.2)  135 |
| Bladder control  Median  Range  Mean (SD)  No. of patients | 0  0-100  12.9 (26.0)  267 | 0  0-100  10.0 (20.5)  134 | 0  0-100  11.9 (24.3)  401 | N/A | 0  0-100  9.6 (21.5)  135 |

- *SD = Standard deviation; no = number*

**Supplementary Table 2. Mean and median changes in health-related quality of life scores for the predefined scales of the EORTC QLQ-C30 and QLQ-BN20 questionnaires at the moment of progression when compared to baseline.**

| **Baseline HRQoL scores** | **Bevacizumab + Lomustine**  **(n=92)** | **Lomustine**  **(n=31)** | **All patients (n=123)** |
| --- | --- | --- | --- |
| *EORTC QLQ-C30* |  |  |  |
| Global health status  Median  Range  Mean (SD)  No. of patients | 0.0  -83.3 to 41.7  -7.7 (23.0)  87 | 0.0  -50 to 25  -8.0 (19.3)  24 | 0.0  -83.3 to 41.7  -7.7 (22.1)  111 |
| Physical functioning  Median  Range  Mean (SD)  No. of patients | -6.7  -100 to 26.7  -102 (21.5)  87 | 0.0  -60 to 20  -8.2 (17.5)  27 | -6.7  -100 to 26.7  -9.7 (20.6)  114 |
| Social functioning  Median  Range  Mean (SD)  No. of patients | 0.0  -100 to 83.3  -1.6 (30.2)  85 | 0.0  -50 to 50  0.0 (18.3)  26 | 0.0  -100 to 83.3  -1.20 (27.8)  111 |
| *EORTC QLQ-BN20* |  |  |  |
| Motor deficits  Median  Range  Mean (SD)  No. of patients | 0.0  -44.4 to 100  3.1 (24.1)  84 | 5.6  -11.1 to 22.2  7.5 (10.1)  26 | 0.0  -44.4 to 100  4.1 (21.6)  110 |
| Communication deficit  Median  Range  Mean (SD)  No. of patients | 0.0  -55.6 to 77.8  2.91 (23.4)  84 | 0.0  -22.2 to 33.3  4.3 (13.4)  26 | 0.0  -55.6 to 77.8  3.23 (21.4)  110 |

- *SD = standard deviation; no = number*

**Supplementary Table 3. Effect of severe toxicity defined as grade 3-5 toxicity on the selected health-related quality of life scales. Patients with and without grade 3-5 toxicity at week 12 was compared.**

|  | **Severe Toxicity (Grade 3-5)** | |  |  |
| --- | --- | --- | --- | --- |
| **Week 12 HRQoL score** | **Present**  **(n=50)** | **Absent**  **(n=125)** | **Missing**  **(n=44)** | **Wilcoxon**  **P-value** |
| *EORTC QLQ-C30* |  |  |  |  |
| Global health status  Median  Range  Mean (SD)  No. of patients | 58.3  0.0 to 83.3  56.5 (19.3)  50 | 66.7  0.0 to 100  65.6 (20.5)  123 | 75.0  33.3 to 100  71.0 (17.7)  42 | 0.0098 |
| Physical functioning  Median  Range  Mean (SD)  No. of patients | 70.0  6.7 to 100  66.5 (26.7)  50 | 80.0  0.0 to 100  76.6 (20.9)  125 | 86.7  0.0 to 100  80.2 (22.4)  43 | 0.0227 |
| Social functioning  Median  Range  Mean (SD)  No. of patients | 83.3  0 to 100  67.3 (29.4)  50 | 83.3  0.0 to 100  71.2 (28.7)  124 | 100   1. to 100   79.5 (30.6)  43 | 0.39 |
| *EORTC QLQ-BN20* |  |  |  |  |
| Motor deficits  Median  Range  Mean (SD)  No. of patients | 19.4  0.0 to 77.8  25.0 (24.0)  50 | 11.1  0.0 to 100  19.2 (21.1)  121 | 0.0  0.0 to 88.9  15.3 (22.2)  43 | 0.14 |
| Comm. deficit  Median  Range  Mean (SD)  No. of patients | 11.1  0.0 to 100  24.5 (26.4)  49 | 11.1  0.0 to 100  23.4 (28.0)  121 | 11.1  0.0 to 100  23.3 (31.6)  43 | 0.58 |

- *SD = standard deviation; no = number*

**Supplementary Table 4. Summary statistics for patients with and without HRQoL forms**

|  | **With HRQoL data**  **(n=415)** | | **Without HRQoL data**  **(n=22)** | | **Total**  **(n=437)** |
| --- | --- | --- | --- | --- | --- |
| **Baseline characteristics** | **Bevacizumab + lomustine**  **(n=275)** | **Lomustine alone**  **(n=140)** | **Bevacizumab + lomustine**  **(n=13)** | **Lomustine alone**  **(n=9)** |  |
| Age, years  Median  Range | 57.3  23.1 – 82.3 | 59.4  21.2 – 79.2 | 56.9  43.2 – 76.7 | 62.6  38.0 – 66.1 | 57.7  21.2 - 82.3 |
| Sex, no. (%)  Male  Female | 164 (59.6)  111 (40.4) | 86 (61.4)  54 (38.6) | 10 (76.9)  3 (23.1) | 5 (55.6)  4 (44.4) | 265 (60.6)  172 (39.4) |
| WHO PS, no. (%)  0  1  2 | 97 (35.3)  155 (56.4)  23 (8.4) | 45 (32.1)  79 (56.4)  16 (11.4) | 3 (23.1)  5 (38.5)  5 (38.5) | 4 (44.4)  2 (22.2)  3 (33.3) | 149 (34.1)  241 (55.1)  47 (10.8) |
| MGMT status, no. (%)  Methylated  Unmethylated  Undetermined/missing | 65 (23.6)  86 (31.3)  124 (45.1) | 36 (25.7)  37 (26.4)  67 (47.9) | 2 (15.4  1 (7.7) 10 (76.9) | 1 (11.1)  1 (11.1)  7 (77.8) | 104 (23.8)  125 (28.6)  208 (47.6) |
| Corticosteroid therapy at trial entry, no. (%) |  |  |  |  |  |
| No | 138 (50.2) | 74 (52.9) | 6 (46.2) | 4 (44.4) | 222 (50.8) |
| Yes | 137 (49.8) | 66 (47.1) | 7 (53.8) | 5 (55.6) | 215 (49.2) |
| Antiseizure medication, no. (%) |  |  |  |  |  |
| No | 101 (36.7) | 43 (30.7) | 4 (30.8) | 2 (22.2) | 150 (34.3) |
| Non-EIAED | 168 (61.1) | 95 (67.9) | 7 (53.8) | 7 (77.8) | 277 (63.4) |
| Switch >2 weeks | 4 (1.5) | 2 (1.4) | 0 (0.0) | 0 (0.0) | 6 (1.4) |
| Switch ≤2 weeks | 1 (0.4) | 0 (0.0) | 1 (7.7) | 0 (0.0) | 2 (0.5) |
| EIAED | 1 (0.4) | 0 (0.0) | 1 (7.7) | 0 (0.0) | 2 (0.5) |
| Resection for recurrence, no. (%) |  |  |  |  |  |
| No | 216 (78.5) | 114 (81.4) | 13 (100.0) | 7 (77.8) | 350 (80.1) |
| Yes | 59 (21.5) | 26 (18.6) | 0 (0.0) | 2 (22.2) | 87 (19.9) |

**WHO = World Health Organization; MGMT = O6-Methylguanine Methyltransferase; EIAED= enzyme-inducing antiepileptic drugs; no = number*

**Supplementary Figure 1: Time to health-related quality of life deterioration (TTQD) and deterioration free survival (QDFS) for each health-related quality of life scale.**

*Global Health status*


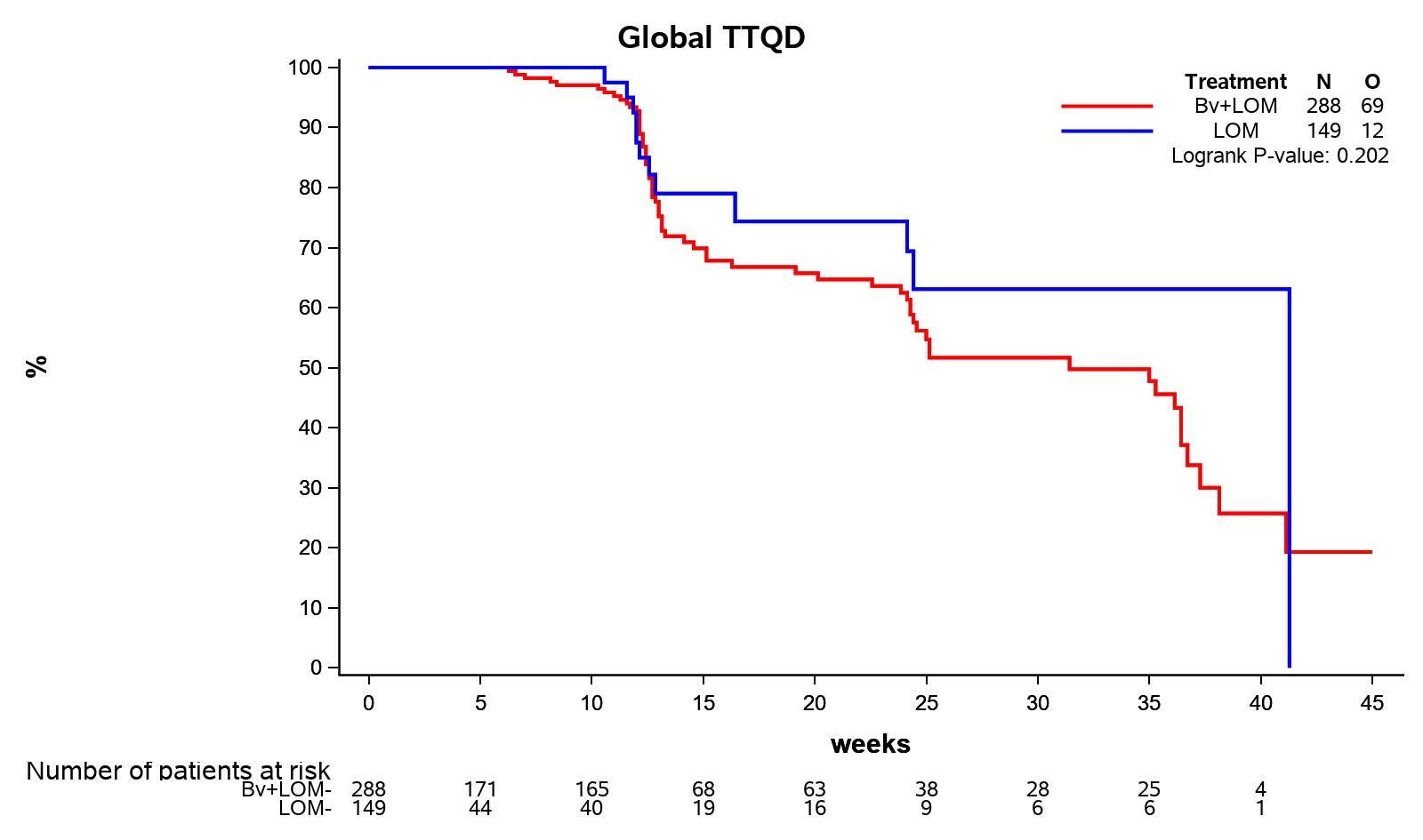


| **Global TTQD** | | | **Non-parametric** | | **Cox model** | |
| --- | --- | --- | --- | --- | --- | --- |
| **Treatment** | **Patients (N)** | **Observed Events (O)** | **Median (95% CI) (Weeks)** | **% at 0.5 Year(s) (95% CI)** | **Hazard Ratio (95% CI)** | **P-Value (Score test)** |
| Bv+LOM | 288 | 69 | 31.43 (24.29, 36.43) | 51.7 (41.3, 61.1) | 1.00 | 0.205 |
| LOM | 149 | 12 | 41.29 (24.14, 41.29) | 63.1 (40.7, 79.0) | 0.67 (0.36, 1.25) |  |
|  |  |  |  |  | Log-rank test: | p-value=0.202 |


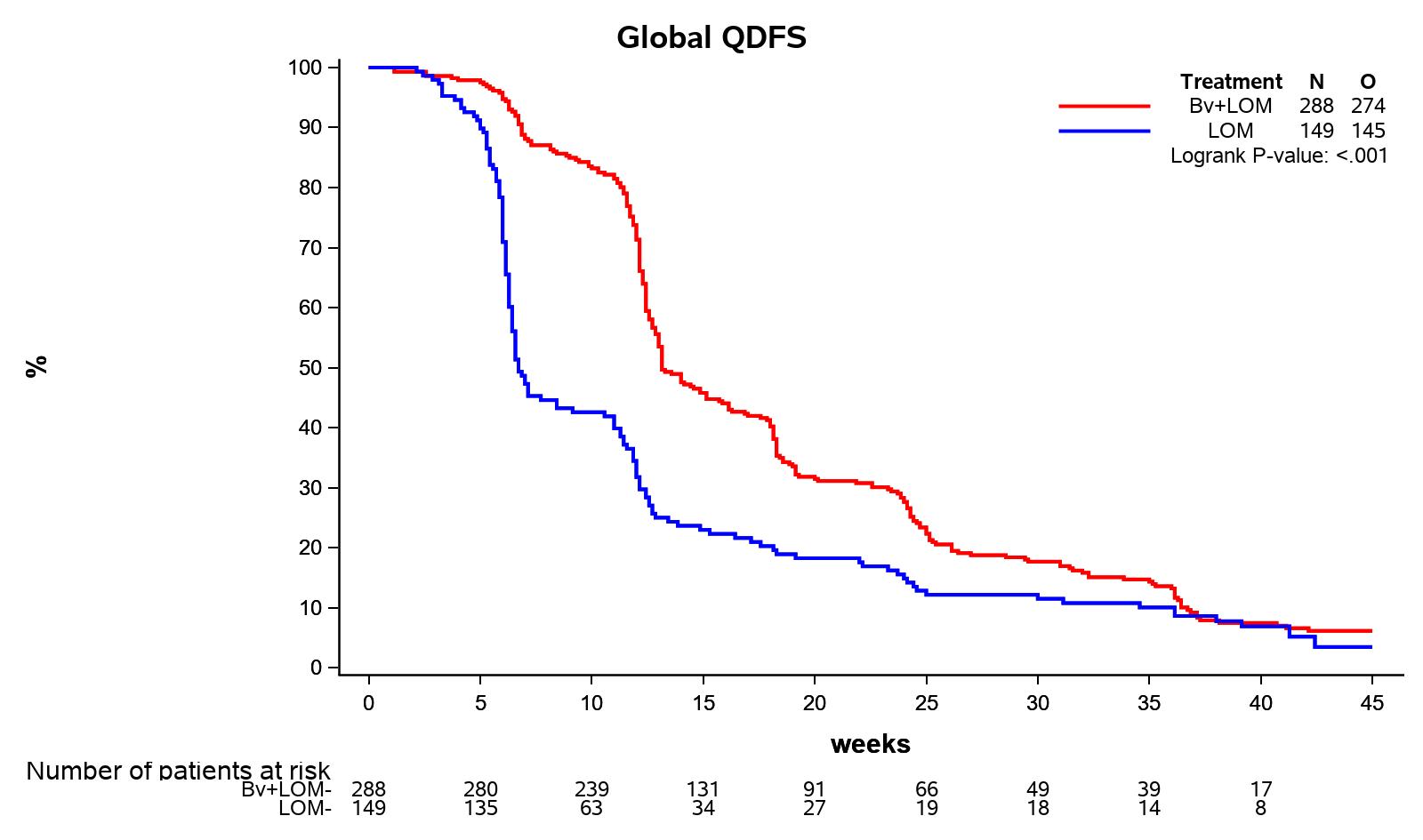


| **Global QDFS** | | | **Non-parametric** | | **Cox model** | |
| --- | --- | --- | --- | --- | --- | --- |
| **Treatment** | **Patients (N)** | **Observed Events (O)** | **Median (95% CI) (Weeks)** | **% at 0.5 Year(s) (95% CI)** | **Hazard Ratio (95% CI)** | **P-Value (Score test)** |
| Bv+LOM | 288 | 274 | 13.14 (12.86, 15.86) | 20.6 (16.1, 25.4) | 1.00 | 0.000 |
| LOM | 149 | 145 | 6.71 (6.43, 10.57) | 12.2 (7.5, 18.0) | 1.76 (1.44, 2.16) |  |
|  |  |  |  |  | Log-rank test: | p-value=0.000 |

*Physical functioning (PF)*


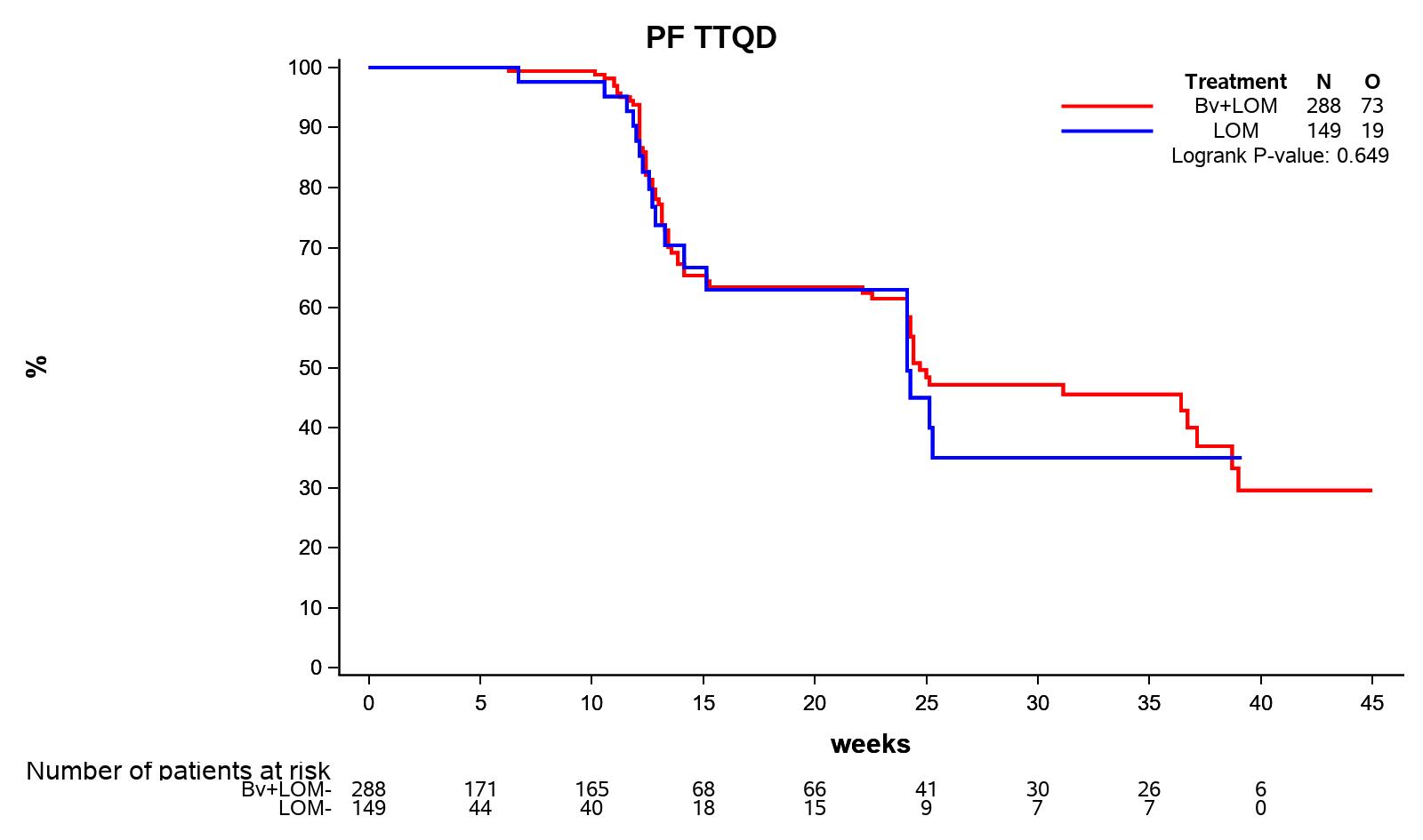


| **PF TTQD** | | | **Non-parametric** | | **Cox model** | |
| --- | --- | --- | --- | --- | --- | --- |
| **Treatment** | **Patients (N)** | **Observed Events (O)** | **Median (95% CI) (Weeks)** | **% at 0.5 Year(s) (95% CI)** | **Hazard Ratio (95% CI)** | **P-Value (Score test)** |
| Bv+LOM | 288 | 73 | 24.71 (24.14, 37.14) | 47.2 (37.4, 56.3) | 1.00 | 0.653 |
| LOM | 149 | 19 | 24.14 (14.14, N) | 35.0 (17.0, 53.6) | 1.12 (0.68, 1.87) |  |
|  |  |  |  |  | Log-rank test: | p-value=0.649 |


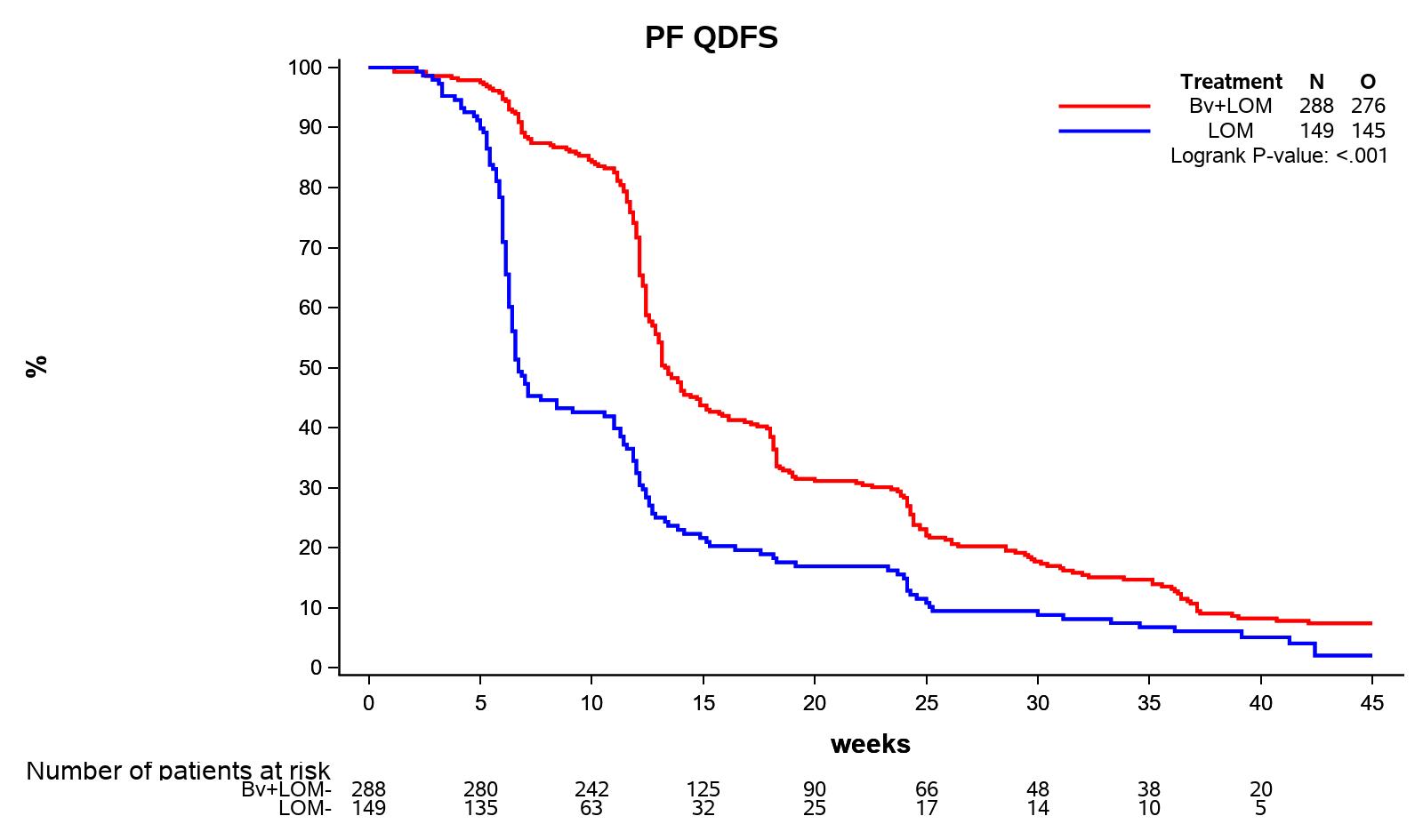


| **PF QDFS** | | | **Non-parametric** | | **Cox model** | |
| --- | --- | --- | --- | --- | --- | --- |
| **Treatment** | **Patients (N)** | **Observed Events (O)** | **Median (95% CI) (Weeks)** | **% at 0.5 Year(s) (95% CI)** | **Hazard Ratio (95% CI)** | **P-Value (Score test)** |
| Bv+LOM | 288 | 276 | 13.36 (12.86, 14.86) | 21.3 (16.8, 26.2) | 1.00 | 0.000 |
| LOM | 149 | 145 | 6.71 (6.43, 10.57) | 9.5 (5.4, 14.8) | 1.93 (1.57, 2.37) |  |
|  |  |  |  |  | Log-rank test: | p-value=0.000 |

*Social functioning (SF)*


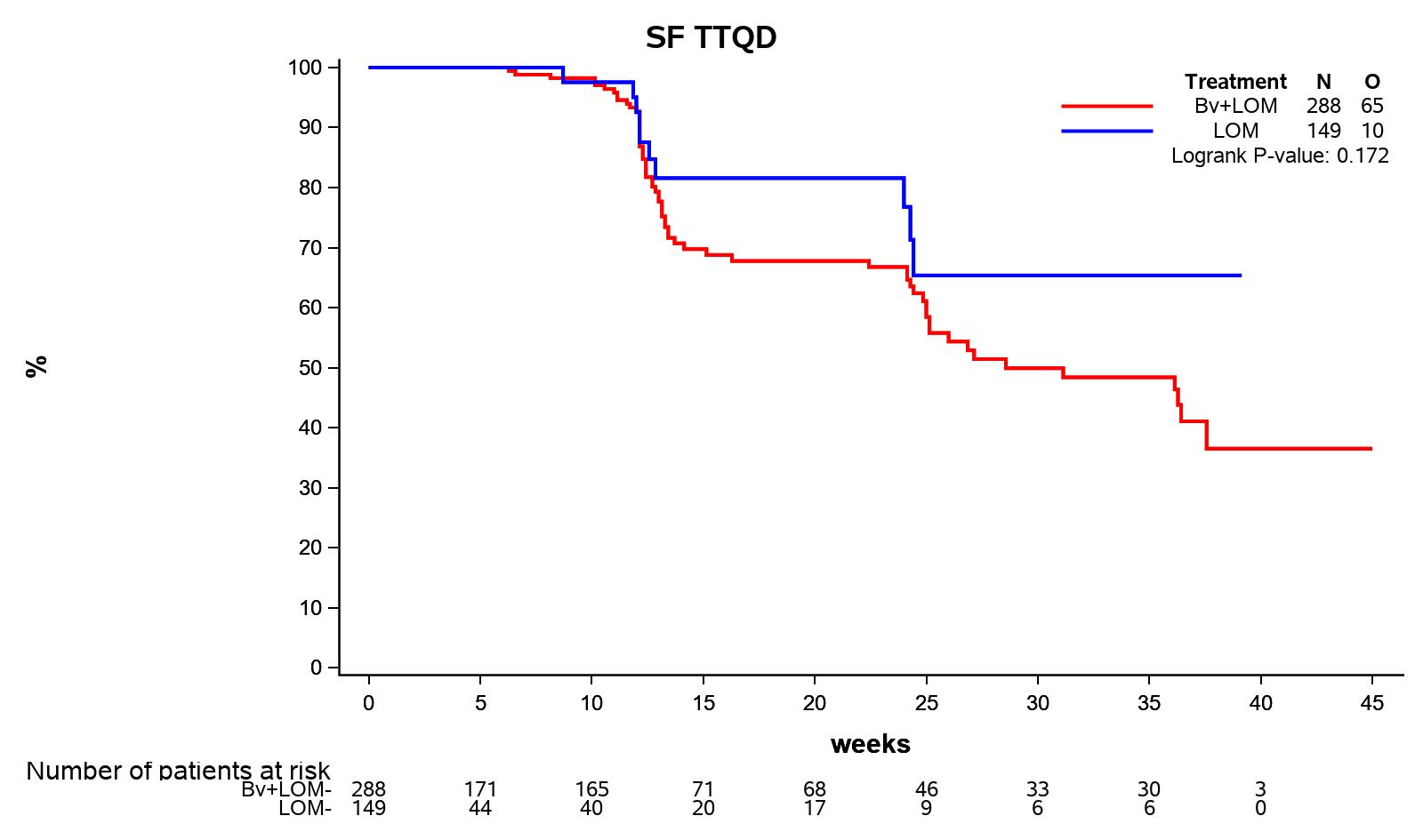


| **SF TTQD** | | | **Non-parametric** | | **Cox model** | |
| --- | --- | --- | --- | --- | --- | --- |
| **Treatment** | **Patients (N)** | **Observed Events (O)** | **Median (95% CI) (Weeks)** | **% at 0.5 Year(s) (95% CI)** | **Hazard Ratio (95% CI)** | **P-Value (Score test)** |
| Bv+LOM | 288 | 65 | 28.57 (25.00, 37.57) | 54.4 (44.2, 63.4) | 1.00 | 0.175 |
| LOM | 149 | 10 | Not reached | 65.4 (42.6, 80.9) | 0.63 (0.32, 1.23) |  |
|  |  |  |  |  | Log-rank test: | p-value=0.172 |


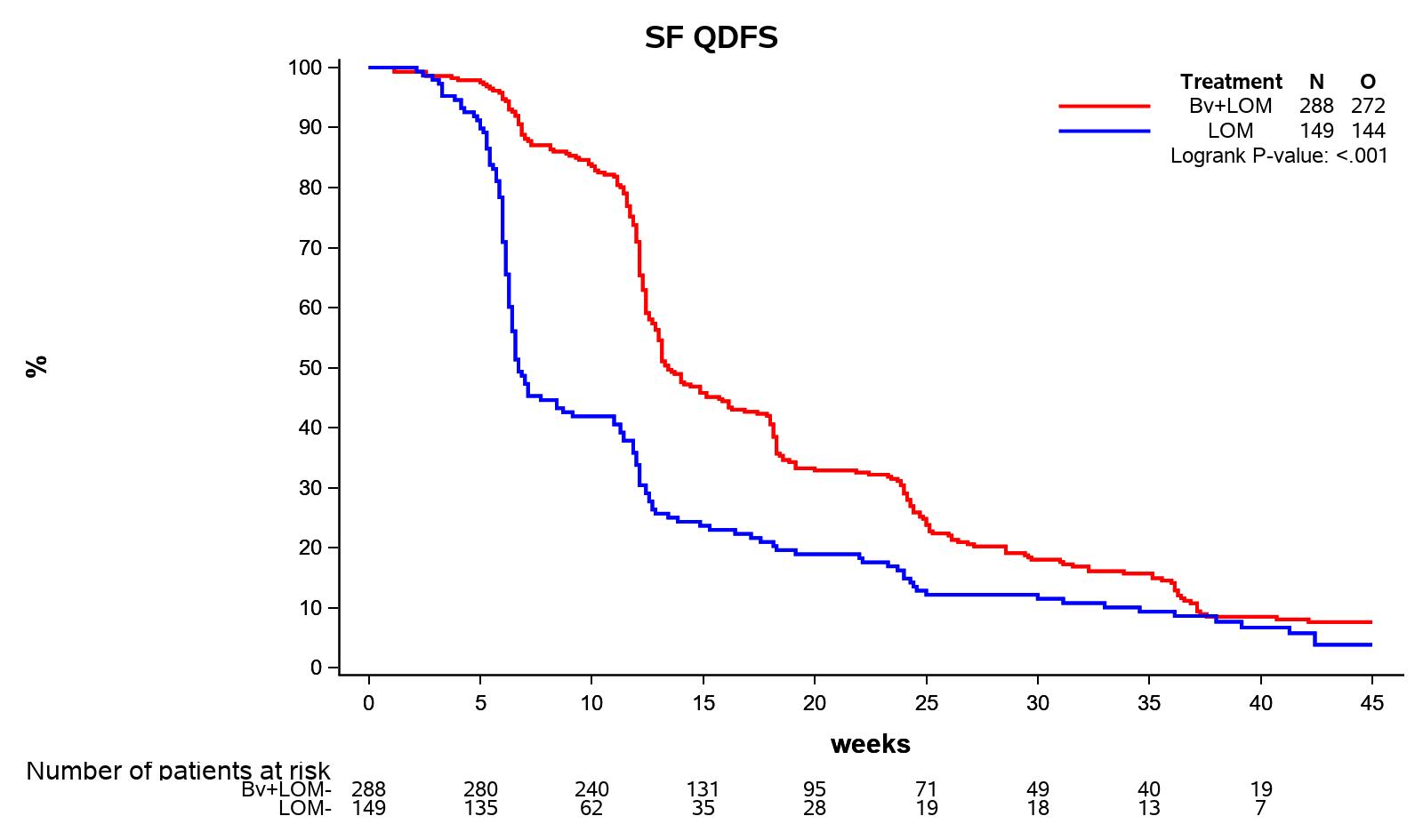


| **SF QDFS** | | | **Non-parametric** | | **Cox model** | |
| --- | --- | --- | --- | --- | --- | --- |
| **Treatment** | **Patients (N)** | **Observed Events (O)** | **Median (95% CI) (Weeks)** | **% at 0.5 Year(s) (95% CI)** | **Hazard Ratio (95% CI)** | **P-Value (Score test)** |
| Bv+LOM | 288 | 272 | 13.43 (13.00, 16.14) | 22.0 (17.4, 27.0) | 1.00 | 0.000 |
| LOM | 149 | 144 | 6.71 (6.43, 9.14) | 12.2 (7.5, 18.0) | 1.80 (1.46, 2.20) |  |
|  |  |  |  |  | Log-rank test: | p-value=0.000 |

*Motor dysfunction (BMD)*


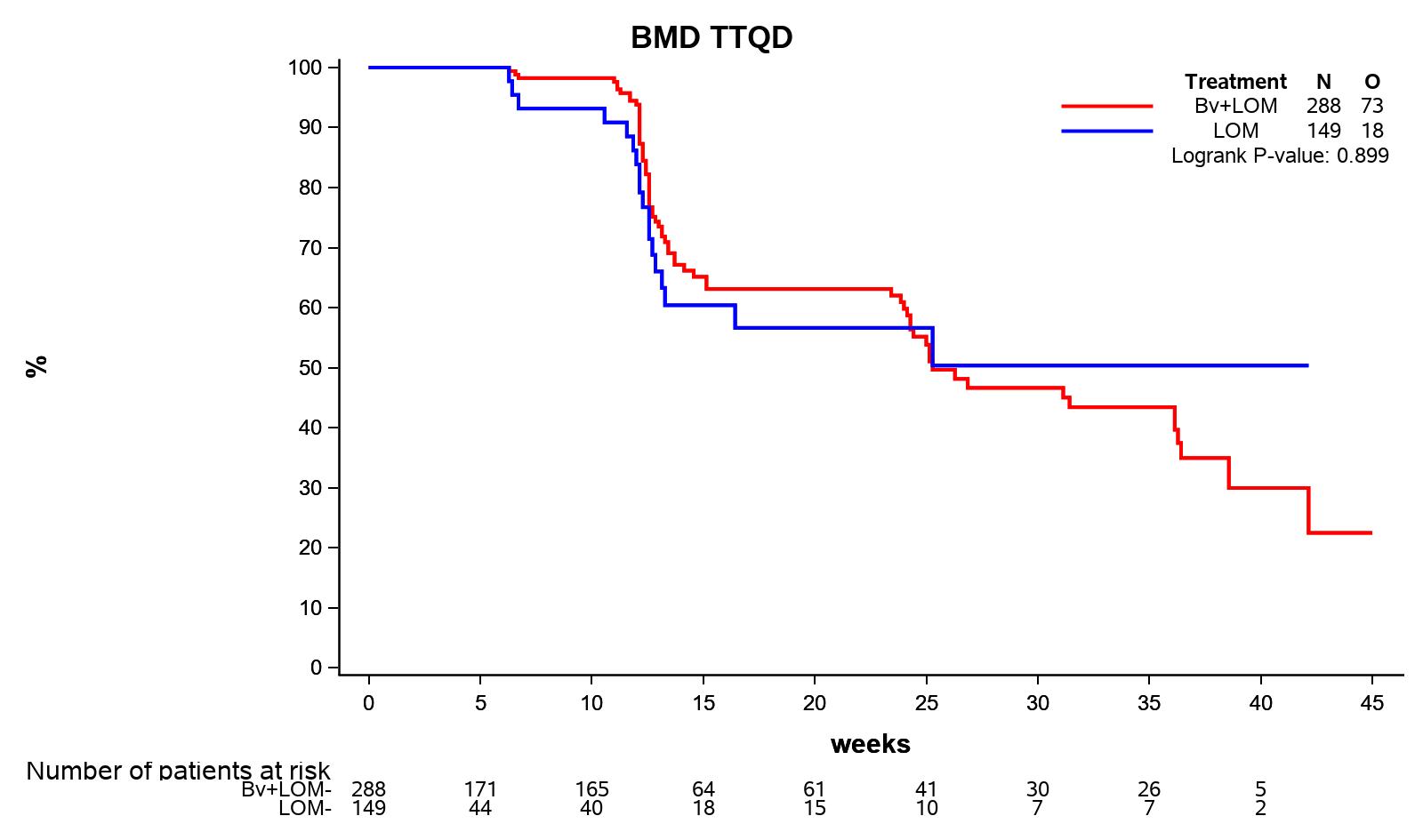


| **BMD TTQD** | | | **Non-parametric** | | **Cox model** | |
| --- | --- | --- | --- | --- | --- | --- |
| **Treatment** | **Patients (N)** | **Observed Events (O)** | **Median (95% CI) (Weeks)** | **% at 0.5 Year(s) (95% CI)** | **Hazard Ratio (95% CI)** | **P-Value (Score test)** |
| Bv+LOM | 288 | 73 | 25.29 (24.14, 36.29) | 49.6 (39.5, 59.0) | 1.00 | 0.900 |
| LOM | 149 | 18 | Not reached | 50.3 (31.1, 66.8) | 0.97 (0.58, 1.63) |  |
|  |  |  |  |  | Log-rank test: | p-value=0.899 |


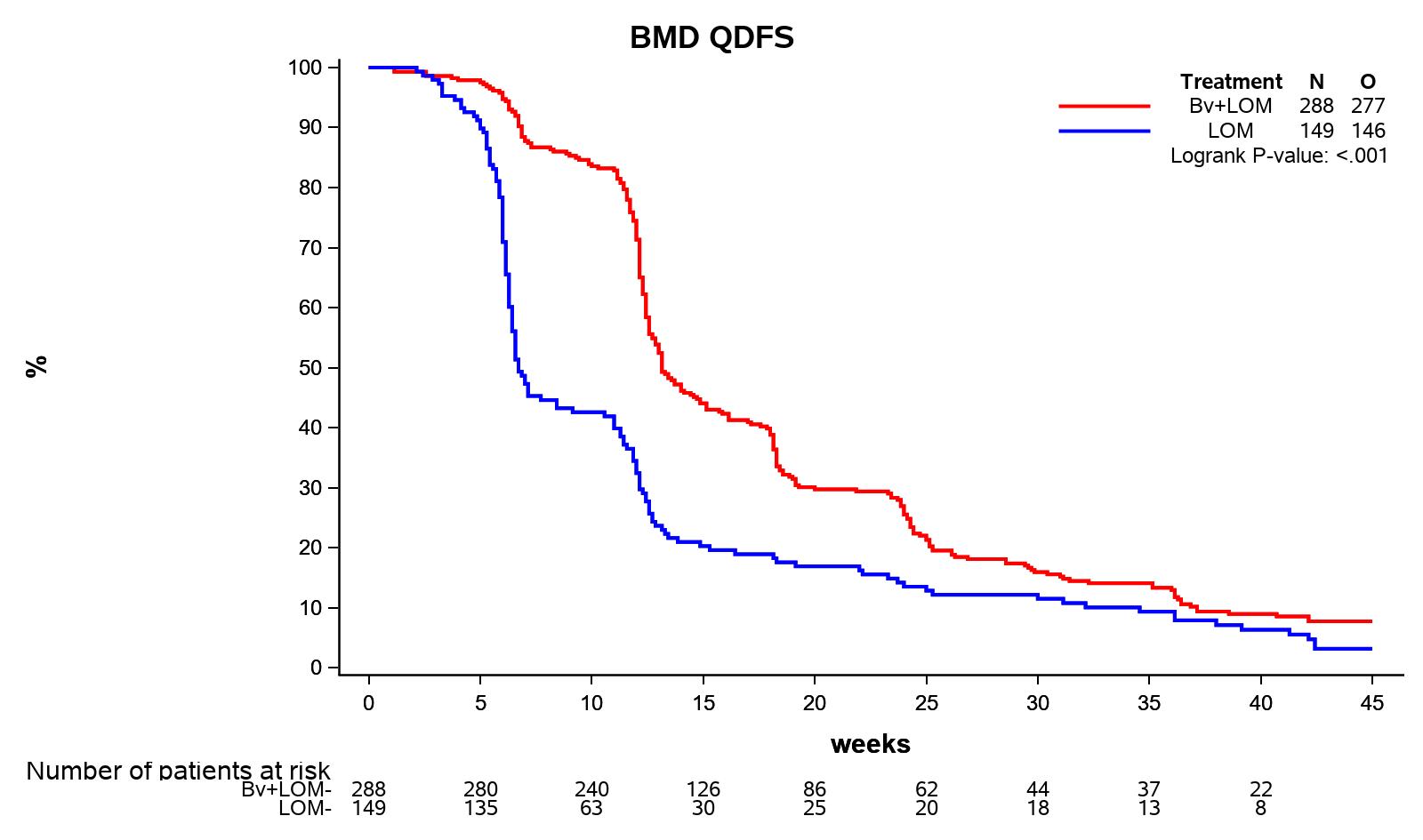


| **BMD QDFS** | | | **Non-parametric** | | **Cox model** | |
| --- | --- | --- | --- | --- | --- | --- |
| **Treatment** | **Patients (N)** | **Observed Events (O)** | **Median (95% CI) (Weeks)** | **% at 0.5 Year(s) (95% CI)** | **Hazard Ratio (95% CI)** | **P-Value (Score test)** |
| Bv+LOM | 288 | 277 | 13.14 (12.57, 14.86) | 19.5 (15.2, 24.3) | 1.00 | 0.000 |
| LOM | 149 | 146 | 6.71 (6.43, 10.57) | 12.2 (7.5, 18.0) | 1.84 (1.50, 2.25) |  |
|  |  |  |  |  | Log-rank test: | p-value=0.000 |

*Communication deficit (BCD)*


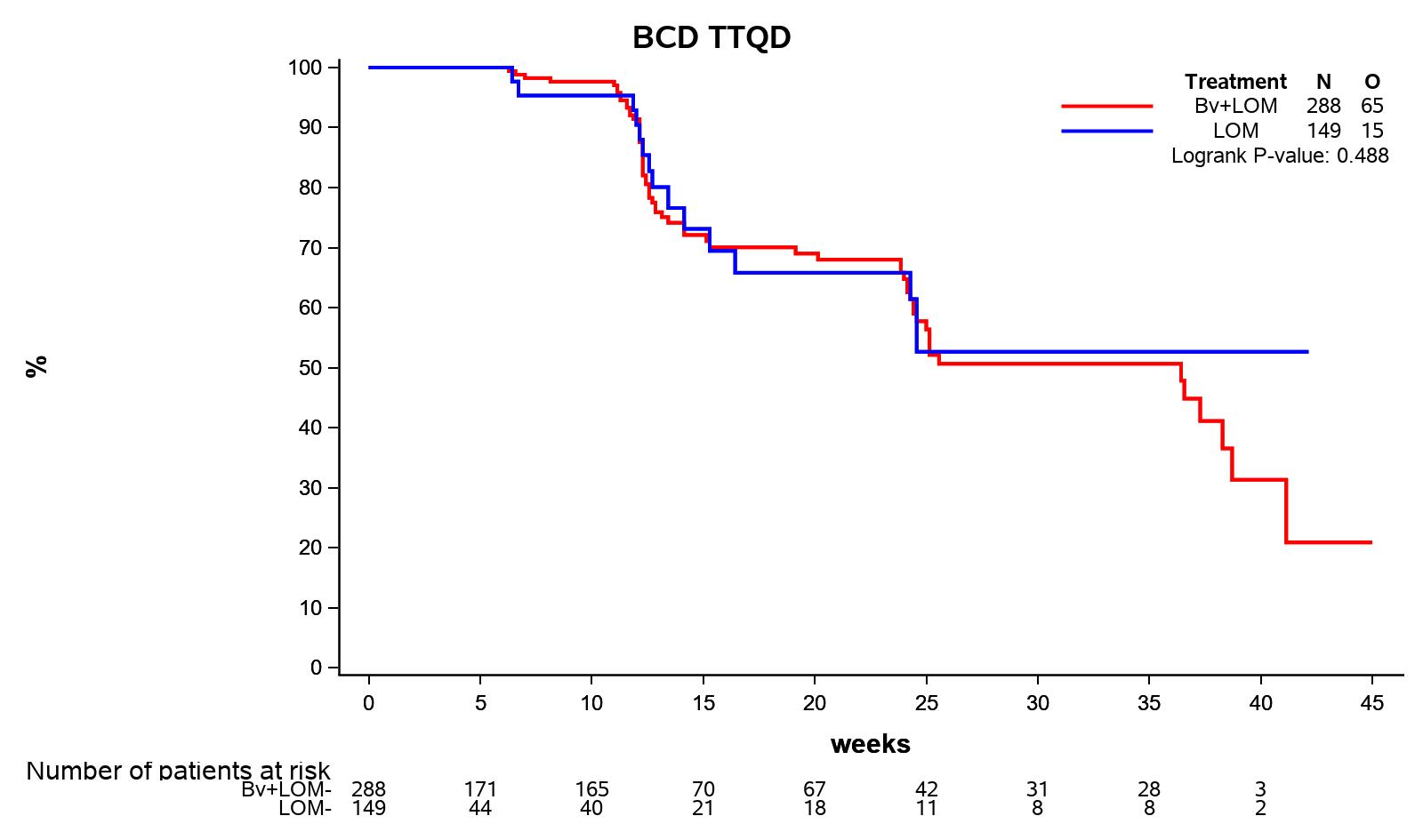


| **BCD TTQD** | | | **Non-parametric** | | **Cox model** | |
| --- | --- | --- | --- | --- | --- | --- |
| **Treatment** | **Patients (N)** | **Observed Events (O)** | **Median (95% CI) (Weeks)** | **% at 0.5 Year(s) (95% CI)** | **Hazard Ratio (95% CI)** | **P-Value (Score test)** |
| Bv+LOM | 288 | 65 | 36.43 (24.43, 38.71) | 50.7 (40.3, 60.1) | 1.00 | 0.492 |
| LOM | 149 | 15 | Not reached | 52.7 (32.8, 69.1) | 0.82 (0.47, 1.44) |  |
|  |  |  |  |  | Log-rank test: | p-value=0.488 |


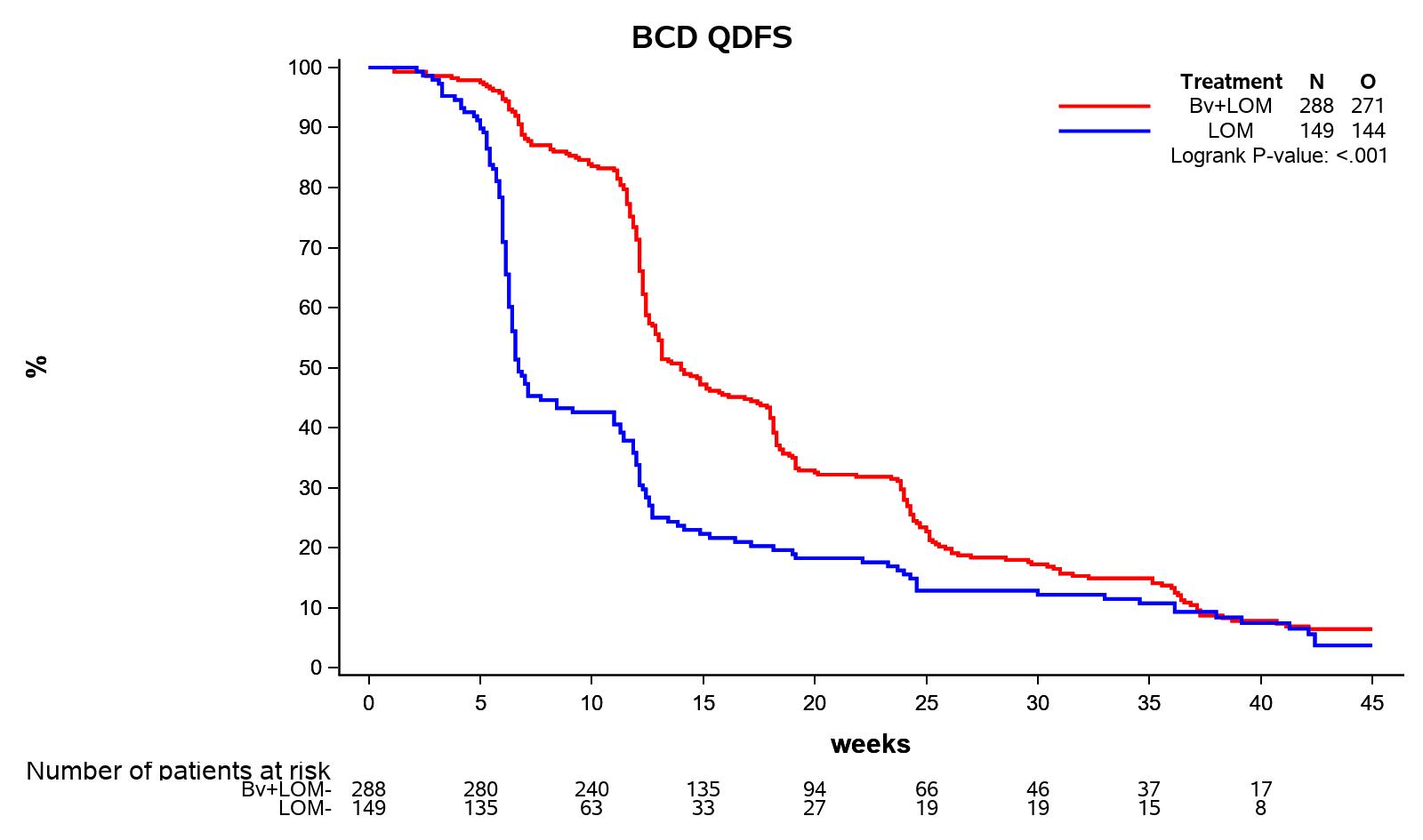


| **BCD QDFS** | | | **Non-parametric** | | **Cox model** | |
| --- | --- | --- | --- | --- | --- | --- |
| **Treatment** | **Patients (N)** | **Observed Events (O)** | **Median (95% CI) (Weeks)** | **% at 0.5 Year(s) (95% CI)** | **Hazard Ratio (95% CI)** | **P-Value (Score test)** |
| Bv+LOM | 288 | 271 | 14.00 (12.86, 17.43) | 19.8 (15.4, 24.6) | 1.00 | 0.000 |
| LOM | 149 | 144 | 6.71 (6.43, 11.00) | 12.8 (8.1, 18.8) | 1.76 (1.44, 2.16) |  |
|  |  |  |  |  | Log-rank test: | p-value=0.000 |

**Supplementary figures 2: Mean changes in scores from baseline for exploratory scales 2A-2Y) separately for bevacizumab plus lomustine and lomustine alone. Differences in scores ≥10 points are considered clinically relevant.**

***2A:*** *Global health status*

***2B****: Physical Functioning*

***2C****: Role Functioning*

***2D****: Emotional Functioning*

***2E****: Cognitive Functioning*

***2F****: Social Functioning*

***2G****: Fatigue*

***2H****: Nausea/ Vomiting*

***2I****: Pain*

***2J****: Dyspnea*

***2K****: Insomnia*

***2L****: Appetite loss*

***2M****: Constipation*

***2N****: Diarrhea*

***2O****: Future uncertainty*

***2P****: Visual Disorder*

***2Q****: Motor dysfunction*

***2R****: Communication deficit*

***2S:*** *Headaches*

***2T****: Seizures*

***2U****: Drowsiness*

***2V****: Hair loss*

***2W****: Itchy skin*

***2X****: Weakness legs*

***2Y****: Bladder control*
